# TB Stigma:  Stigma and Stigmatization Experienced by People with Tuberculosis: A Qualitative Study in Indonesia

**DOI:** 10.12688/f1000research.177509.1

**Published:** 2026-07-10

**Authors:** Aan Sutandi, Henny Suzana Mediani, Apriana Rahmawati, Sari Narulita, Maryuni Maryuni, Fatma Refaat Ahmed, Nabeel Al-Yateem, Muhammad Arsyad Subu, Richard Mottershead

**Affiliations:** 1 Faculty of Nursing and Midwifery, Universitas Binawan, Jakarta, Indonesia; 2 School of Nursing, Padjadjaran University, Bandung, Indonesia; 3 Faculty of Nursing, Jember University, Java, Indonesia; 4 Faculty of Nursing, College of Health Sciences, University of Sharjah, Sharjah, United Arab Emirates; 5 Behavioural Science Institute, SEHA, Sakina, Al Ain, United Arab Emirates; 6 College of Nursing, University of Baghdad, Baghdad, Iraq

**Keywords:** stigma, stigmatization, tuberculosis, TB, qualitative study, Indonesia

## Abstract

**Background:**

Tuberculosis (TB) disease significantly impacts society’s physical, financial, and social well-being. TB stigma can have a severe effect on individuals and families and lead to social withdrawal or isolation.

**Aims:**

This study explored the stigma faced by TB patients in two urban areas of Jakarta, Indonesia.

**Methods:**

This study adopted a descriptive qualitative design. Purposive sampling was used to recruit study participants. We conducted semi-structured interviews with 30 participants at a hospital and a public health center in Indonesia. We analyzed the data using a content analysis approach.

**Results:**

Our study identified four themes associated with tuberculosis’s stigma: 1) internalized stigma or self-stigma, 2) family stigma, 3) public/social stigma, and 4) the reasons for TB stigma. TB transmission and control behaviors were influenced in certain instances by stigma-induced non-disclosure. Stigma-alleviating treatments are necessary to prevent prejudice and protect patient confidentiality. These interventions include education campaigns about tuberculosis treatment and transmission.

**Conclusion:**

Tuberculosis stigma was primarily experienced as social isolation and avoidance motivated by apprehension regarding contagion, rumours, and verbal abuse. To reduce the misconceptions surrounding TB, stigma-reduction strategies, such as community awareness programs and the establishment of social support groups, may increase treatment adherence and service utilization.

## Introduction

Tuberculosis (TB) is a contagious illness that primarily impacts the respiratory system. However, it can also target other areas of the body. Excessive population density in residential or occupational settings is a significant contributing factor to the spread of tuberculosis, and only individuals who are afflicted with tuberculosis have the potential to transmit the disease ( World Health Organization [WHO], 2024a). TB is a significant global health concern that engenders various physical, economic, and social issues ( DeSanto et al., 2023). Globally, about 10.6 million individuals contracted TB in 2021 ( WHO, 2023). US$22 billion is required annually for TB prevention, diagnosis, treatment, and care to meet global targets by 2027, as agreed at the 2023 UN High-Level TB meeting ( WHO, 2025). China, Bangladesh, Nigeria, the Congo, India, Pakistan, Indonesia, and the Philippines together represented more than two-thirds of the total tuberculosis cases worldwide ( WHO, 2023). Malnutrition was identified as the cause of 2.2 million new cases of tuberculosis worldwide. Alcohol use disorder was responsible for 740,000 new cases, while smoking was related to 690,000 cases ( WHO, 2023). Tuberculosis (TB) can impact individuals of all demographics and geographical locations. However, this disease primarily affects adults, with a higher prevalence among males than women ( WHO, 2022). Although TB is a disease that may be prevented and treated, it still causes the deaths of 1.5 million people annually, making it the leading cause of infectious mortality worldwide. TB is the primary cause of death in people with HIV and is a significant contributor to the development of antibiotic resistance worldwide ( WHO, 2024a).

In Indonesia, TB poses a significant public health challenge. Managing it is a central goal in the country’s efforts to promote sustainable healthcare. The country reported approximately 150,000 TB cases, equating to one case every four minutes. In 2020, TB caused 93,000 deaths, with a mortality rate of 55 per 100,000 people ( Ministry of Health of Indonesia, 2022a). Indonesia is among eight nations that account for two-thirds of global TB cases, according to the WHO Global TB Report 2020. In 2019, an estimated 142,000 children were diagnosed with TB, representing 17% of all TB cases in Indonesia. Effective TB control can enhance population quality and productivity ( Ministry of Health of Indonesia, 2023). The 2018 Indonesian Basic Health Research reported a TB prevalence of 0.4%, comparable to the 2013 figure. Provinces with the highest prevalence were Banten (0.8%), Papua (0.8%), West Java (0.6%), and Aceh (0.5%) ( Hadawiyah et al., 2022). The Ministry of Health estimated that in 2023, Indonesia had about 969,000 TB cases, resulting in 144,000 TB-related deaths, including 28,000 with drug-resistant TB and 7,921 confirmed RR/MDR TB cases. Of these deaths, 13,977 were due to TB ( Ministry of Health of Indonesia, 2023). TB remains one of Indonesia’s top five causes of death, hampering efforts for complete detection, which was only 41% in 2020, nearly halving from previous levels ( Ministry of Health of Indonesia, 2022b). Most TB risk factors are linked to poor living conditions, poverty, and socioeconomic inequality ( Al-Qarana & Sfenrianto, 2022). In Indonesia, TB is closely associated with poverty and economic hardship, with sufferers often facing stigma and discrimination.

Erving Goffman (1963) described stigma as an attribute that deeply discredits and diminishes an individual, transforming them from a complete, typical person into a tainted, marginalized one (p. 3). Both internal and external stigma hinder TB patients and survivors from exercising their rights to access healthcare ( Noya, 2022). In DKI Jakarta, Indonesia, TB remains one of the most prevalent diseases among the population. In 2018, there were 39,470 cases of pulmonary TB in the city, with the highest numbers in East Jakarta (12,334) and South Jakarta (7,859). Other districts reported 7,563 in West Jakarta, 6,428 in Central Jakarta, 5,215 in North Jakarta, and 71 in the Seribu Islands ( Badan Pusat Statistik Province DKI Jakarta, 2022). Social stigma is a significant factor contributing to the worsening of pulmonary tuberculosis, as inappropriate behavior can continue to expose others to the disease ( Zulaikhah et al., 2019).

The extent of stigmatization depends heavily on cultural and contextual value systems, which vary across regions and societies ( Ahmedani, 2011; Subu et al., 2023). TB stigma often marks those affected by the disease, and public health communication about tuberculosis has been inconsistent, sometimes even unintentionally fueling stigma ( Meershoek et al., 2018; Subu et al., 2021). Using a conceptual framework on health-related stigma can help unify terminology and provide deeper insights into how stigmatization functions ( Stangl et al., 2019). TB remains a highly stigmatized disease ( Datiko et al., 2020; Thomas & Stephen, 2021). The stigma is linked to its transmission, limited knowledge about its cause, treatment, or association with marginalized groups like those with low income, racial minorities, sex workers, or HIV/AIDS ( Ministry of Health of Indonesia, 2022a). Stigma affects TB treatment programs, particularly by causing patients to abandon treatment, which is the main challenge in managing pulmonary TB ( Endria & Yona, 2019). One factor is the social stigma that discourages seeking treatment, as TB symptoms such as coughing and weight loss often attract negative attitudes from social circles ( Oladele et al., 2021). This stigma can lead to social isolation, rejection, or exclusion from work, community, family, and even healthcare providers trusted with their care ( Pradipta et al., 2021). Much of this stigma stems from false beliefs and misconceptions, like the idea that TB is incurable, hereditary, a curse, or spread through non-respiratory means such as sharing utensils. Some TB stigmas may be based on legitimate public health concerns about transmission ( Nyasulu et al., 2016; Chang & Cataldo, 2014). However, stigma can hinder treatment adherence, reducing effectiveness and raising the risk of the disease spreading and becoming drug-resistant ( Datiko et al., 2020; Sommerland et al., 2017). Broadly, evidence suggests that TB stigma can contribute to mental health issues ( Lee et al., 2017), financial hardship, and income loss ( Zimmerman et al., 2022). Given these issues, it is crucial to study TB stigma to understand its prevalence, causes, and determinants, and to assess the success of strategies aimed at reducing it ( Sommerland et al., 2017).

Socially, TB can lead to various issues such as poverty, stigma, and discrimination. Research shows that individuals who internalize stigma often have low self-esteem, and fear of adverse community reactions can prevent them from disclosing their status ( Hadawiyah et al., 2022). This qualitative study examined the stigma and discrimination faced by people with TB from their own perspectives in two urban Indonesian areas. Understanding what contributes to TB stigma can help improve efforts to identify, treat, and engage people with the disease. These insights are also vital for designing effective TB treatment programs and interventions that address this issue specifically in Indonesia.

## Methods

### Design

The main research objective of this study was to explore the stigma and stigmatization experienced by people with tuberculosis in two urban areas in Indonesia. Qualitative research is a type of research that examines the fundamental nature and significance of the world by employing interpretive and naturalistic approaches. We conducted a qualitative content analysis, which involved assessing the topic and context of the collected data and identifying contrasts and similarities within and between the different data sources. Content analysis examines distinctions and similarities across text sections, with particular emphasis on subject matter and context ( Graneheim et al., 2017).

### Setting and participants

The research was conducted at Esnawan Antariksa Hospital, Halim Perdanakusuma Jakarta, and the District Public Health Center (PHC) in East Jakarta between July and November 2023. Purposive sampling was used to recruit 30 study participants. We conducted semi-structured interviews with 30 participants with TB who were on treatment at a hospital and a public health center in Jakarta, Indonesia.

### Data collection

Semi-structured interviews were the primary method used for data collection. The interviews with patient participants lasted between 35 and 55 minutes. Supplementary data was gathered through memos, field notes, and document reviews. Using these supplementary data-collection methods facilitated data triangulation, enhancing the reliability of the data interpretations. Additionally, we conducted a comprehensive review of digital and physical records available within the hospital and the PHC. These mute data were crucial to shaping our understanding of the participants’ experiences, attitudes, and beliefs. We conducted 30 semi-structured interviews in Indonesian. To ensure uniformity across interviews, a group of seasoned interviewers (MAS, SR, and MM) and local research team members conducted all interviews. Before the interview, each participant confirmed that they had carefully read the participant information sheet and thoroughly understood the study. Before conducting the interview, informed consent was obtained. The interviews were conducted in the hospital and Primary Health Care (PHC) conference rooms, as well as in nurses’ offices. During the interviews, participants were given sufficient time for reflection to prevent any sense of pressure in their responses. After the interview, participants were allowed to ask additional questions.

### Data analysis

We employed inductive content analysis ( Graneheim & Lundman, 2004) to extract broad themes from a selected subset of transcripts. One research method for identifying specific words, themes, or concepts within each set of qualitative data is content analysis (i.e., text analysis). Through content analysis, researchers can measure and examine the frequency, significance, and connections between specific terms, themes, or ideas ( Graneheim et al., 2017). This approach was appropriate for our research because our goal was to acquire a comprehensive comprehension of the encounters of Indonesian patients with various types of TB stigma. In this study, we developed themes through the iterative process of carefully reading and manually assigning codes. As part of the analysis process, we sometimes reviewed the interview transcripts to gain a deep understanding of the content. Then, we systematically combined and labeled the words, phrases, and paragraphs, considering their interconnectedness in terms of the content and context of stigma. Next, sections about the personal experiences of the participants on various forms of TB stigma were isolated and stored in a distinct text document. The codes and units of meaning were analyzed within the study’s context and evaluated for similarities and differences. The final codebook was implemented in QSR NVivo to manage all transcripts, including those manually coded. This process resulted in the finalization of themes and the collection of supporting quotations. Finally, themes aligned with the stigmas described in the literature were established.

### Ethical considerations

In this study, all participants provided written consent before participating. Study approval was received from the University of Binawan Health Research Ethics Committee (No. 033/KEPK-UBN/I/2023). Before agreeing to participate in the interview, participants were provided with a comprehensive description of the study. Additionally, participants were informed that they had the right to withdraw from the study without any unfavorable consequences. All participants provided written informed consent prior to participating in the research.

## Results

This study employed semi-structured interviews with 30 tuberculosis patients in Jakarta, Indonesia. Of the 30 participants, 21 were female, and nine were male. Our study identified four main themes: 1) internalized stigma or self-stigma, 2) family stigma, 3) public stigma, and 4) the reason for TB stigmatization. Additionally, each of these central themes has several subthemes. Themes and main themes are discussed below:

### Theme 1: Internalized stigma or self-stigma

The study revealed that participants had internalized the negative emotions they experienced, known as internalized stigma or self-stigma.

***Subtheme: Status loss***

Participants expressed experiencing shame and feeling alienated from the community, as well as being perceived as distinct from other individuals considered to be typical. For example, a male TB patient expressed that he was insulted and humiliated:

*Yes, I experience a status loss … shame. In society, insult and discredit exist* … *I am insulted. I am considered a TB patient and humiliated. I have been offended. I am sad, and my heart sobs uncontrollably* … *(Participant 4)*.

***Subtheme: Rejection and avoidance***

Patients with TB also had the belief that they were subjected to rejection and avoidance due to their condition. Participants stated that community members rejected them due to societal misconceptions about TB. One participant said:

*Indeed, those afflicted with tuberculosis experience social rejection. The community disapproves of this illness. It appears that only a few people can accept our condition without rejection. Additionally, a problem is that tuberculosis causes avoidance … I've noticed that others tend to avoid my presence … (Participant 15).*

***Subtheme: Discrimination***

A female participant described incidents of discrimination she faced due to her tuberculosis (TB) diagnosis. She said:

… *I experience discrimination* … *We [patients with TB] are often subjected to unfair treatment compared to individuals with other medical conditions, such as hypertension and diabetes. Yes, I am a victim of discrimination. (Participant 25).*

### Theme 2: Family sigma

Study participants expressed concerns about their perspectives and personal experiences with familial stigma. Stigma negatively affects people with TB because of stigma among their family members.

***Subtheme: Lack of family support***

Family support is crucial to the recovery of an individual with tuberculosis. However, our study participants reported that due to stigma and humiliation. Their family members offered/little assistance and support.

*… My family members do not provide support for me. Occasionally, because of my condition, my parents experience feelings of humiliation* … *my father and mother do not support me. Because … yes, they [family members] experience a feeling of shame (Participant 10)*.

***Subtheme: Family rejection***

Study participants also reported that relatives afflicted with tuberculosis were frequently rejected by their extended families. This rejection reportedly occurred despite the hospitalization of a patient with tuberculosis.

*Yes, they are typically rejected* … *Families may find it particularly challenging to convince long-term TB patients to accept their relatives. Because they believe that a person with TB will be disgraced. Then, an individual with TB is rejected or refused (Participant 16)*.

### Theme 3: Public/social stigma

***Subtheme: Social labelling***

The consequence of a tuberculosis diagnosis is labelling. A participant recounted the difficulties he encountered in securing employment because of the tuberculosis label attached to him.

*… I am stigmatized or labelled by my society* … *I am labelled as a TB … or other as an ex-TB person. Then, if I look for employment, it is challenging for me* … *(Participant 22).*

***Subtheme: Limited social support***

Social support is crucial for TB patients to achieve better outcomes. The study participants emphasized public stigma. They reported that members of their communities subjected them to public stigma, thereby limiting community support. Participants reported that most tuberculosis patients lacked this social support.

*Support from them (people in the community) is limited. Fully true* … *I do not have any form of support* … *I have no support whatsoever. Members of the community only have concerns with themselves. No, I do not get support from others at this time. They want me to leave* … *(Participant 21)*.

***Subtheme: Social isolation***

Some participants raised concerns regarding situations of community isolation directed at people who have tuberculosis. A tuberculosis patient described their personal experience of social isolation within their community.

*Yes … without a doubt, this remains there. They isolated me [members of the community]. Consequently, I was angry and had no desire to interact with the residents of my community. I continue to experience [the desire for] vengeance due to my rejection and isolation (Participant 7)*.

***Subtheme: Lack of social acceptance***

Participants stated that patients with TB experienced a lack of social acceptance or rejection. A participant described the repercussions of individuals with tuberculosis being marginalized within their society.

*People with tuberculosis were excluded from society due to societal rejection. Others reject them. They will avoid individuals who are afflicted. Individuals experience fear because of this [TB]* … *(Participant 4).*

***Subtheme: Social discrimination***

Most participants reported significant discrimination associated with tuberculosis. For example, individuals diagnosed with tuberculosis were denied re-entry into their previous place of employment due to their tuberculosis status. A participant said:

*Individuals are discriminated against due to tuberculosis. Yes, it is true* … *discrimination. Employers are unwilling to rehire an individual with tuberculosis in his or her previous position because he or she is ex-tuberculosis … (Participant 12).*

### Theme 5: Reasons for TB stigmatization

***Subtheme: Lack of knowledge and understanding***

Study participants identified limited knowledge of the cause (including misconceptions regarding the hereditary nature of tuberculosis), mode of transmission, treatment, and the failure of a cure as potential factors contributing to stigmatization.

*Regardless of the extent of treatment administered or the announcement of recovery, people will continue to avoid him out of concern that they may transmit the disease to another. It is because of a lack of knowledge. They do not understand that TB can be cured …* ( *Participant 9).*

***Subtheme: Public’s fear***

Study participants indicated that people had a sense of fear towards individuals with tuberculosis because they believed that they [other people] would be at risk of contracting tuberculosis.

*The community members are experiencing fear, sir* … *feeling fearful, frightened, or apprehensive. Yes, society is fearful. Indeed, individuals often experience fear and tend to flee from threatening situations. Due to societal fear, those suffering from TB are often neglected (Participant 25).*

***Subtheme: Fear of TB transmission to others***

The participants in the study were concerned about the possibility of contracting the disease and its severe consequences.

*… I choose to be alone. I do not like being with another person* … *She [a mother] informs me not to be close to a small child because she is at risk of contracting TB. Yes, I chose to withdraw from them. I might infect them … (Participant 11).*

***Subtheme: Fear of being associated with HIV/AIDS***

Some study participants and their family members expressed apprehension about acquiring and/or being recognized as having HIV/AIDS.

*I was not encouraged by family members to undergo HIV testing, as they would blame me. You know, if someone discovered he was positive [HIV-AIDS] [unclear] … HIV is among the most feared diseases among individuals (Participant 15).*

## Discussion

This study aimed to explore the stigma faced by people diagnosed with tuberculosis (TB) in an urban area of Indonesia. TB continues to be a significant global health issue, especially in Indonesia, with high rates of cases and deaths. A total of 856,000 people contracted TB, leading to 84,000 deaths. These figures represent 8% of the worldwide cases and 7% of global TB-related deaths ( WHO, 2024b).

Research on TB stigma and its impacts has been documented ( Ali, 2019; Subu et al, 2024). TB stigma primarily manifests as social exclusion and avoidance, fueled by fears of transmission, rumors, verbal harassment, diminished marriage prospects, and family neglect ( Mukerji & Turan, 2018). In India, TB is often stigmatized because it challenges societal norms that define acceptability. Patients often face enacted and perceived stigma, while their family members encounter perceived and secondary stigma. Healthcare workers can also display secondary stigma ( Abbas Ali et al., 2024). Stigmatization arises from beliefs, attitudes, and behaviors targeting individuals or groups perceived as stigmatized ( Stangl et al., 2019). The community setting plays a significant role in shaping TB-related stigma. Although well-recognized, it is increasingly studied within public health research ( MacIntyre et al., 2017). TB stigma can hinder efforts to seek and stick to treatment ( Cremers et al., 2015; Krishnan et al., 2014). Stigma involves devaluing or discrediting individuals because of undesirable traits, often through labeling, marginalization, stereotyping, and social exclusion based on those traits ( Chang & Cataldo, 2014; Krishnan et al., 2014).

Our research shows that stigma appears both openly and internally. Self-stigma, or internalized stigma, involves accepting others’ criticism. Similar studies in Africa find that internalized stigma lowers self-esteem, while anticipated stigma prevents people from revealing their status out of fear of negative consequences ( Cremers et al., 2015; Murray et al., 2013). People experiencing internalized stigma often have reduced self-esteem ( Daftary et al., 2021; Wouters et al., 2020). Our results confirm that stigma is present, predictable, and internalized. Literature documents stigma among TB patients and healthcare workers, whether expected, enacted, or internalized ( Foster et al., 2022; MacIntyre et al., 2017). TB patients also experience feelings of inferiority linked to self-stigma, often showing signs like avoiding eye contact or looking down during conversations. This internalized stigma stems from feelings of isolation, disrespect, and insignificance ( Ritsher et al., 2003; Saraswati et al., 2016). TB-related stigma is driven by fear of catching the disease and the dual understanding of its nature. People with TB try to avoid social stigma associated with seeking treatment, which hinders access to care. Stigma in TB patients can lead to discrimination, withdrawal, or self-isolation ( Fuady et al., 2023). Studies indicate that internalized stigma causes feelings of guilt and lowers self-esteem, leading to poorer treatment adherence ( Atre et al., 2011; Cremers et al., 2015). The literature consistently reports internalized stigma among TB patients and healthcare staff across different contexts ( MacIntyre et al., 2017; Foster et al., 2022). Some African research shows internalized stigma diminishes self-worth, while anticipated stigma affects willingness to disclose status due to fear of negative reactions ( Murray et al., 2013; Cremers et al., 2015). Additionally, individuals with internalized stigma tend to have low self-esteem ( Wouters et al., 2020; Daftary et al., 2021).

People affected by TB-related stigma, including their families, have indicated that guidance from TB survivors on effective recovery methods would be helpful. TB survivors serve as living proof that TB and its social stigma can be overcome, which can improve treatment adherence and ensure ongoing medical support ( DeSanto et al., 2023). Stigma involves perceptions of others’ judgments, fear of disclosure, internalized shame and self-rejection, self-stigmatization, and real experiences like community or household exclusion, discrimination, and harmful behaviors ( Stangl et al., 2019; Sotgiu & Dobler, 2020).

Stigma impacts not only individuals but also entire families. Our study found TB stigma exists among family members of TB patients in Indonesia. Understanding ‘family stigma’ is essential to reduce social and emotional distress and to prevent families from avoiding support and treatment ( Park & Park, 2014). Such stigma can hinder individuals from accessing TB care, resulting in delays, poorer treatment outcomes, and increased transmission risk within families and communities ( Datiko et al., 2020; Soemarko et al., 2023). Within families, TB stigma may appear as lack of support, isolation, or shame toward a member with TB ( Ngurah et al., 2018). People with TB often face social ostracism from friends and relatives. Similar to our findings, TB patients face significant stigma from family and friends ( Kamble et al., 2020). One study showed that married women are sometimes prejudged, experiencing neglect from family members who expect them to fulfill traditional caregiving roles even when unwell ( Mukerji & Turan, 2018). Lay, Manurung, and Landi (2021) identified that family stigma stems from a lack of knowledge about TB—its causes, transmission, and treatment. Misconceptions lead to mistreatment, such as believing TB can be caught from talking or sharing towels. However, education and counseling from healthcare professionals help change perceptions, fostering recognition of TB as a serious disease. Addressing TB stigma effectively involves seeking care at health facilities ( Lay et al., 2021).

Family support involves help from immediate family members—such as spouses, children, siblings, and parents—for individuals with tuberculosis. Such support makes patients feel recognized and cared for, helping them to complete their treatment without neglect or abandonment ( Lay et al., 2021). TB was once viewed as a family disease because of its extensive impact on all family members. The stigma linked to TB originates from its perception as an unclean illness ( Fuady et al., 2023). Much of this shame appears to result from a lack of understanding about tuberculosis.

Social or public stigma involves prejudice, discrimination, or stereotypes that diminish an individual’s self-esteem by promoting negative attitudes or marginalizing them ( Ali, 2019; Hadawiyah et al., 2022). This study found that TB patients face stigma in social and public contexts. Consistent with other research, societal stigma adversely impacts TB patients ( Hariadi et al., 2023; Stangl et al., 2019). TB influences social interactions and is linked to high community stigma, which can discourage patients from seeking or sticking to treatment. Stigma can be driven not only by patients’ own choices but also by societal attitudes and health workers blaming TB patients for their condition, thereby reinforcing community stigma ( Datiko et al., 2020). Our findings agree with previous studies showing that TB patients experience significant stigma stemming from environmental factors and fears of transmission ( Rizqiyah, 2021). A common negative perception is that TB is brought by the patient and can be transmitted, with many viewing it as a cursed disease ( Sajodin et al., 2022). Patients often feel unable to socialize and become isolated to avoid insults, gossip, or rejection ( Fuady et al., 2023; Sajodin et al., 2022). Literature already documents various aspects of TB stigma, including social exclusion, prejudice, and discrimination ( Mohammedhussein et al., 2020; Karat et al., 2021). Beyond dealing with symptoms and side effects, many patients also face financial hardship due to reduced or lost income, insecure living conditions, social isolation, mental health issues, and strained relationships ( Karat et al., 2021).

Social support is essential for improving outcomes among TB patients. Our study indicates that one key factor behind TB stigma is a lack of knowledge and understanding within families and communities. These factors include individuals’ knowledge levels and psychosocial aspects such as risk perception and fear of infection ( Muhidin et al., 2020). Awareness about TB significantly affects how people behave towards those with the disease ( Hariadi et al., 2023). The fear influences not only TB patients but also those around them, leading to concerns such as fear of contracting or transmitting the disease, delays in diagnosis, and treatment nonadherence ( Courtwright & Turner, 2010; Craig et al., 2017). Stigma sources also include fears related to disease, transmission, and death. Feelings of stigma from genuine threats or perceived community attitudes may result in discrimination based on various traits, illnesses, or societal factors ( Hariadi et al., 2023). The fear of infection and of spreading TB has been widely documented in studies exploring TB-related stigma in different contexts ( Chang & Cataldo, 2014; Sommerland et al., 2017).

### Study limitation

A limitation of this study is its small sample size, consisting of only two tuberculosis health facilities in Jakarta, Indonesia. Consequently, caution should be exercised when generalizing these findings to other settings. However, both patients and former patients share their experiences prior to seeking medical help, highlighting factors that foster tuberculosis-related stigma. Further research involving patients from various TB healthcare environments could help validate these findings.

## Conclusion

In this study, we found that stigma mediation was highly personalized and depended on an individual’s understanding and personal experiences with stigma. This was evident in the differing perspectives of community members, with some believing that stigma labeling occurred at facilities, while others believed it occurred through ward-based outreach teams. People’s perceptions of stigma significantly influenced their use of healthcare services, including a preference for receiving care at home rather than in a healthcare institution or for avoiding care altogether. We also found that one factor contributing to TB stigma is a lack of knowledge and understanding among families and communities. Further study is essential to develop a comprehensive understanding of how to address TB stigma effectively and responsibly, as interventions have the potential to inadvertently perpetuate stigmatization.

## Data Availability

Participant data contains sensitive personal information, and sharing such data publicly could compromise confidentiality and anonymity. The Institutional Review Board (IRB) has mandated that data sharing is permissible only under specific conditions that ensure participant privacy and align with ethical guidelines. Access to the data may be granted to qualified researchers for legitimate academic purposes upon request. Requests for access must be submitted in writing to the corresponding author Dr. Aan Sutandi − aan@binawan.ac.id
