# TB Stigma and Stigmatization Experienced by People with Tuberculosis: A Qualitative Study in Jakarta, Indonesia

**DOI:** 10.12688/f1000research.177509.2

**Published:** 2026-09-17

**Authors:** Aan Sutandi, Henny Suzana Mediani, Apriana Rahmawati, Sari Narulita, Maryuni Maryuni, Fatma Refaat Ahmed, Nabeel Al-Yateem, Muhammad Arsyad Subu, Richard Mottershead

**Affiliations:** 1 Faculty of Nursing and Midwifery, Universitas Binawan, Jakarta, Indonesia; 2 School of Nursing, Padjadjaran University, Bandung, Indonesia; 3 Faculty of Nursing, Jember University, Java, Indonesia; 4 Faculty of Nursing, College of Health Sciences, University of Sharjah, Sharjah, United Arab Emirates; 5 Behavioural Science Institute, SEHA, Sakina, Al Ain, United Arab Emirates; 6 College of Nursing, University of Baghdad, Baghdad, Iraq

**Keywords:** stigma, stigmatization, tuberculosis, TB, qualitative study, Indonesia

## Abstract

**Background:**

Tuberculosis (TB) remains a major global public health challenge, with Indonesia accounting for a substantial proportion of global incident disease. Although previous Indonesian studies have demonstrated the prevalence and consequences of TB-related stigma, less is known about how people receiving TB treatment experience the interconnected processes of internalised, family and public/social stigma, and how they understand the factors driving stigmatization in everyday life.

**Aim:**

This study explored stigma and stigmatization experienced by people with TB receiving treatment in two urban healthcare settings in Jakarta, Indonesia.

**Methods:**

This study used a qualitative descriptive design. Purposive sampling was used to recruit 30 adults receiving TB treatment from a hospital and a public health centre in Jakarta, Indonesia. Data were generated through individual semi-structured interviews conducted in Bahasa Indonesia and analysed using inductive qualitative content analysis.

**Results:**

Four interrelated themes were identified: (1) internalised stigma or self-stigma; (2) family stigma; (3) public/social stigma; and (4) reasons for TB stigmatization. Participants described shame and loss of social status, reduced family support, social labelling, isolation and discrimination, including consequences for employment. Stigmatization was attributed particularly to limited knowledge and misconceptions about TB, fear of infection and transmission, and association with HIV/AIDS.

**Conclusion:**

TB stigma in these Jakarta settings operated across individual, family and community levels and could remain socially consequential during treatment and anticipated recovery. Stigma-reduction strategies should therefore extend beyond education alone and combine accurate communication about transmission and curability with psychosocial and peer support, family and community engagement, and measures to address social and employment discrimination.

## Introduction

Despite major advances in prevention, diagnosis and treatment, tuberculosis (TB) remains one of the world’s most consequential infectious diseases. The World Health Organization (WHO) estimated that 10.7 million people developed TB in 2024 and 1.23 million died from the disease. Indonesia accounted for approximately 10% of global incident TB, second only to India, and was also among the countries contributing substantially to the global gap between estimated incident disease and notified cases ( World Health Organization [WHO], 2025). TB therefore remains both a biomedical challenge and a marker of persistent inequities in access to timely diagnosis, continuity of care and social protection. The financial implications are equally substantial: global TB commitments require sustained investment in prevention, diagnosis, treatment and care, while affected households may experience catastrophic expenditure, interrupted employment and loss of income ( WHO, 2025; Zimmerman et al., 2022).

These consequences are amplified by the social meanings attached to TB. Stigma can influence whether people recognise symptoms, seek diagnosis, disclose their illness, remain engaged with treatment and participate fully in family and community life ( Courtwright & Turner, 2010; Craig et al., 2017; Datiko et al., 2020). TB-related stigma has been associated with delayed healthcare seeking, concealment of diagnosis, social withdrawal, loss of employment, disrupted relationships and reduced treatment adherence ( Cremers et al., 2015; Karat et al., 2021; Oladele et al., 2021). Its consequences can therefore extend beyond individual distress to affect public-health outcomes, including delayed case detection and continued transmission. A growing body of work consequently argues that ending TB requires attention not only to biomedical care but also to stigma, discrimination and the psychosocial conditions in which treatment occurs ( Foster et al., 2022; Nuttall et al., 2022).

Conceptual clarity is important because stigma and stigmatization are related but not identical. Goffman (1963) conceptualised stigma as a socially discrediting attribute that alters how a person is perceived and positioned within social relationships. Later work emphasised that stigma is not simply an individual characteristic but a social process involving labelling, stereotyping, separation, status loss and discrimination in contexts where power enables those processes to operate ( Link & Phelan, 2001). In this study, stigma refers to the devalued meaning attached to TB and to people identified with the disease, whereas stigmatization refers to the processes through which that devaluation is anticipated, experienced, reproduced or internalised.

The Health Stigma and Discrimination Framework offers a useful lens for understanding these processes across individual, interpersonal, organisational and structural levels ( Stangl et al., 2019). It distinguishes stigma drivers and facilitators from stigma marking, manifestations and outcomes. Within TB, drivers may include fear of infection, misconceptions about transmission, association with poverty or other stigmatised conditions, and moral judgement. Manifestations may include anticipated stigma, enacted discrimination and internalised stigma, while consequences may include avoidance of care, social withdrawal, psychological distress and reduced access to resources ( Stangl et al., 2019). Stigma may also extend to people associated with the affected individual. This associative or secondary stigma is particularly relevant to families, who may experience shame, altered social standing or avoidance because of their relationship to a person with TB ( Park & Park, 2014).

TB creates a distinctive context for stigmatization because genuine concerns about airborne transmission can become entangled with inaccurate or persistent social fears. Infection-prevention behaviours may be medically justified during periods of infectiousness; stigmatization occurs when fear is transferred from a time-limited biological risk to the person as a socially devalued identity. Across settings, misconceptions that TB is incurable, hereditary, morally tainted or transmitted through ordinary social contact have been associated with avoidance, exclusion and discrimination ( Chang & Cataldo, 2014; Lay et al., 2021; Nyasulu et al., 2016). Such responses may continue even after treatment has reduced infectiousness, meaning that social consequences can outlast the biological risk.

The psychosocial consequences of TB stigma are increasingly evident. Internalised and anticipated stigma have been associated with low self-esteem, concealment, social withdrawal and depression ( Cremers et al., 2015; Lee et al., 2017). In Indonesia, Fuady et al. (2024) reported a substantial intersecting burden of TB stigma and depression among adults receiving TB care, with stigma and depression associated with poorer quality of life and significant unmet need for psychosocial support. These findings challenge models of TB care that focus predominantly on medication adherence while giving limited attention to emotional, relational and social recovery.

Indonesia is a particularly important setting in which to examine these experiences. Alongside its high national TB burden, previous Indonesian research has identified barriers to successful treatment at personal, social and health-system levels ( Pradipta et al., 2021). Indonesian studies have also demonstrated measurable TB-related stigma in community and workplace contexts and have documented associations with self-esteem, social relationships and treatment behaviour ( Fuady et al., 2023; Hadawiyah et al., 2022; Soemarko et al., 2023). These studies establish that stigma is present and consequential; however, quantitative measures cannot fully explain how stigmatization unfolds in everyday interactions or how people interpret the responses of relatives, neighbours, employers and others.

Qualitative studies from other high-burden settings similarly identify social rejection, fear of contagion, gossip, diminished marriage prospects, employment discrimination, family strain and concealment as recurrent elements of TB stigma ( DeSanto et al., 2023; Mukerji & Turan, 2018; Oladele et al., 2021). Yet stigma is strongly shaped by cultural and social context, and its manifestations cannot simply be transferred from one setting to another ( Chang & Cataldo, 2014; Dias et al., 2025; Stangl et al., 2019; Subu et al., 2024). In Indonesia, existing work has often quantified stigma, focused on treatment barriers, or examined particular settings such as workplaces. Less is known about how people receiving TB treatment themselves describe the interaction between internalised stigma, family stigma and public/social stigma, and how they explain the mechanisms that produce those experiences in an urban Indonesian context.

This gap is clinically important because interventions will differ depending on the mechanism involved. A knowledge deficit may require improved communication about transmission and curability; anticipated rejection may require confidential, person-centred disclosure support; internalised shame may require psychological or peer support; family stigma may require family-inclusive education; and employment discrimination may require organisational and policy responses. Recent Indonesian intervention development has begun to move in this direction, including co-development of peer-led psychosocial support, individual counselling and community ‘TB Talks’ ( Fuady et al., 2025). Contextually grounded qualitative evidence can help ensure that such interventions address the forms and drivers of stigma that people with TB actually encounter.

Accordingly, this study aimed to explore stigma and stigmatization experienced by people with TB receiving treatment in two healthcare settings in Jakarta, Indonesia. Specifically, it sought to describe the forms of stigma experienced at personal, family and public/social levels and to identify the factors participants perceived as contributing to stigmatization. By centring the perspectives of people receiving TB treatment, the study aimed to generate practice-oriented evidence capable of informing culturally responsive, person-centred TB care and stigma-reduction strategies.

## Methods

### Study design and methodological orientation

This study used a qualitative descriptive design with individual semi-structured interviews. Qualitative description was selected because the aim was to provide a comprehensive, practice-oriented account of participants’ experiences while remaining close to the language and meanings expressed in their narratives. Sandelowski (2000, 2010) argues that qualitative description is particularly appropriate when researchers seek a direct yet analytically meaningful account of health-related experiences rather than the theory generation of grounded theory or the philosophical interpretation associated with phenomenology. The approach is therefore well suited to clinically focused nursing and public health research in which findings are intended to inform service delivery and intervention development.

The methodological design was distinguished from the analytical method. Qualitative description constituted the overarching design, whereas inductive qualitative content analysis provided the analytical strategy. Qualitative content analysis permits systematic examination of meaning units, codes, similarities and differences within textual data and can move from manifest description towards higher levels of abstraction while maintaining traceability to participants’ accounts ( Graneheim & Lundman, 2004; Graneheim et al., 2017). An inductive approach was used because the study sought to derive patterns from participants’ narratives rather than apply a predetermined coding framework ( Elo & Kyngäs, 2008; Hsieh & Shannon, 2005).

### Reporting standards

The revised manuscript was prepared with reference to the Consolidated Criteria for Reporting Qualitative Research (COREQ) and the Standards for Reporting Qualitative Research (SRQR) ( O’Brien et al., 2014; Tong et al., 2007). COREQ is a 32-item framework addressing the research team and reflexivity, study design, and analysis and reporting of interview and focus-group studies. The frameworks were used to strengthen transparency of reporting and were not used to retrospectively attribute procedures that were not undertaken.

### Study setting

The study was conducted between July and November 2023 at Esnawan Antariksa Hospital, Halim Perdanakusuma, Jakarta, and a District Public Health Centre (Puskesmas) in East Jakarta. These settings represent hospital and primary-care components of the urban TB treatment pathway. The study was intentionally situated within these two Jakarta services; the findings are therefore presented as contextually grounded rather than nationally representative.

### Participants, eligibility and sampling

Purposive sampling was used to recruit people with direct experience of TB treatment who could provide information-rich accounts of stigma and stigmatization. Thirty participants were recruited: 12 women (40.0%) and 18 men (60.0%), aged 25–57 years. Most had been living with TB for at least one year and were receiving treatment through either a Puskesmas or hospital. All enrolled participants were in the continuation phase of treatment at the time of interview, and the majority were receiving isoniazid and rifampicin.

Eligible participants were adults aged 18 years or older who had been diagnosed with TB for at least 6 months, were currently receiving treatment or had recently completed treatment, were able to provide informed consent, and had sufficient experience of the treatment trajectory to discuss TB-related stigma. The eligibility framework also allowed inclusion of people who had experienced treatment delay or non-adherence, as well as those who had successfully completed treatment. Exclusion criteria were severe illness requiring immediate hospitalisation, residence in the same household as an already enrolled participant (to maintain independence of accounts), age younger than 18 years, and a TB duration of less than 6 months. Although recently treated completers were eligible, all participants enrolled in the final sample were reported to be in the continuation phase at interview.

Participant demographic and clinical characteristics are summarised in Table 1.

**Table 1.** Participant characteristics. Available participant characteristics are summarised in Table 1. Age, sex, duration of TB experience, treatment setting, treatment phase and medication information were available for the sample. Occupation and living-environment variables were not available and therefore could not be included in the participant profile.

Characteristic	Category/statistic	n (%) or summary
Sex	Women	12 (40.0%)
	Men	18 (60.0%)
Age	Range	25–57 years
Time since TB diagnosis	Duration	Most participants: at least 1 year; eligibility minimum: 6 months
Treatment setting	Care setting	Hospital and Puskesmas both represented
Treatment phase	Status at interview	All participants in continuation phase
Medication regimen	Reported treatment	Majority receiving isoniazid and rifampicin

### Recruitment and consent

Potential participants were approached by the first and second authors (AS and HSM) at Puskesmas facilities, hospitals, or in their homes. Eligibility was assessed against the study criteria before enrolment. Each prospective participant received a participant information sheet and a verbal explanation of the study, was given an opportunity to ask questions, and provided written informed consent before interview. Participation was voluntary, and participants were informed of their right to withdraw without unfavourable consequences.

### Data collection

Data were generated primarily through 30 individual face-to-face semi-structured interviews conducted in Bahasa Indonesia. Interviews lasted approximately 35–55 minutes and took place in private rooms within the hospital and public health centre, including conference rooms and nurses’ offices, with home-based contact used where appropriate during recruitment. Participants were given sufficient time to reflect before responding and were able to ask questions after the interview. Semi-structured interviewing enabled consistent exploration of TB-related stigma while allowing participants to elaborate on personally significant experiences, relationships and meanings.

All interviews were conducted by AS, HSM, NAY and MAS. These researchers had experience in qualitative interviewing and health research. Field notes and research memos were used to capture contextual observations and emerging analytical reflections, and relevant digital and physical records within the hospital and Puskesmas were reviewed to support contextual understanding. These supplementary materials informed interpretation of participants’ experiences but were not treated as substitutes for the interview data.

### Data preparation, transcription, translation and data management

All interviews were audio-recorded and transcribed verbatim. AS, HSM, NAY and MAS conducted the interviews and were responsible for transcription of the interview material. Transcripts were checked against the recordings during data preparation to support accuracy before coding. Data analysis and coding were undertaken in Bahasa Indonesia so that initial interpretation remained as close as possible to participants’ original language and cultural expression.

Following analysis in Bahasa Indonesia, the same four researchers (AS, HSM, NAY and MAS) translated the interview material required for English-language reporting. Cross-language qualitative research may lose nuance when meaning is transferred between languages; accordingly, translation focused on conceptual fidelity rather than literal word-for-word substitution ( van Nes et al., 2010). The translators were fluent in Bahasa Indonesia and English and reviewed wording, terminology and sentence structure to preserve the intended meaning of participants’ accounts. Two additional nurse colleagues with IELTS scores of 7.0 or above independently compared the original Indonesian material with the English translations to assess clarity and fidelity.

### Data analysis

Data were analysed using inductive qualitative content analysis informed primarily by Graneheim and Lundman (2004) and Graneheim et al. (2017), with complementary guidance from Elo and Kyngäs (2008) and Hsieh and Shannon (2005). Analysis was iterative. Transcripts were read repeatedly to develop familiarity with each account and with the dataset as a whole. Text relevant to the study aim was then identified as meaning-bearing segments, including words, phrases, sentences and paragraphs.

Meaning units were examined in context, condensed while retaining their core meaning, and assigned descriptive codes. Codes were compared for similarity and difference and were progressively clustered into more abstract groupings. The analysis moved repeatedly between coded segments, surrounding transcript text and the developing thematic structure to preserve contextual meaning and avoid treating statements as isolated fragments ( Graneheim & Lundman, 2004).

Sections relating to participants’ experiences of different forms of TB stigma were collated in working documents. Codes and meaning units were compared within and across accounts for similarities and differences, and the developing coding framework was refined iteratively. Coding was undertaken in Bahasa Indonesia by four researchers (HSM, SN, MAS and MM). The final codebook was implemented in QSR NVivo 12 to organise and retrieve material across the full dataset, including transcripts that had initially been coded manually. NVivo functioned as a data-management tool; coding decisions, abstraction and interpretation remained researcher-led. Final themes were reviewed and further developed by the full author group, and consensus was reached on the final thematic structure.

The final analysis generated four overarching themes: internalised stigma or self-stigma; family stigma; public/social stigma; and reasons for TB stigmatization. Themes were understood as coherent patterns of meaning rather than simple counts of repeated words or topics. Quotations were selected to demonstrate the empirical basis of the interpretation and are used in the Results to support, rather than substitute for, analytical narrative.

Primary coding was conducted by HSM, SN, MAS and MM. The coding team compared meaning units and codes across transcripts and refined the developing codebook iteratively. Potential differences in interpretation were addressed through collective review of the coded material and continued reference to the source transcripts. The final themes were subsequently reviewed and further developed by the full author group, and consensus was reached on the final thematic structure. An external researcher also reviewed each component of the coded data during the coding process, providing an additional layer of analytical scrutiny.

An illustrative example of the analytical progression from participant meaning units through condensation and coding to subthemes and final themes is presented in Table 2.

**Table 2.** Illustrative analytical pathway. Table 2 provides an illustrative audit trail showing the progression from participant meaning units through condensed meaning and coding to subthemes and final themes. This transparent pathway demonstrates how the higher-order thematic structure remained grounded in participants’ accounts.

Original meaning unit (abridged)	Condensed meaning	Illustrative code	Subtheme	Theme
“I experience a status loss … shame … I am considered a TB patient and humiliated.” (P4)	Diagnosis changes perceived social worth and produces shame.	Shame and diminished social status	Status loss	Internalised stigma/self-stigma
“My family members do not provide support … my parents experience feelings of humiliation.” (P10)	Family shame is accompanied by withdrawal of support.	Family shame and reduced support	Lack of family support	Family stigma
“I am labelled as a TB … or an ex-TB person. Then, if I look for employment, it is challenging.” (P22)	TB identity persists and affects employment opportunities.	Persistent labelling affects employment	Social labelling	Public/social stigma
“They isolated me … I had no desire to interact with the residents of my community.” (P7)	Community exclusion leads to anger and withdrawal.	Exclusion and reactive social withdrawal	Social isolation	Public/social stigma
“They do not understand that TB can be cured.” (P9)	Avoidance is sustained by inaccurate knowledge about curability.	Misconceptions about TB and recovery	Lack of knowledge and understanding	Reasons for TB stigmatization
“I chose to withdraw … I might infect them.” (P11)	Fear of transmitting TB leads the participant to self-isolate.	Fear-driven protective withdrawal	Fear of transmitting TB	Reasons for TB stigmatization

### Trustworthiness and reflexivity

Trustworthiness was addressed through procedures relevant to credibility, auditability/dependability and fittingness/transferability ( Chiovitti & Piran, 2003; Graneheim & Lundman, 2004). Credibility was supported by purposive recruitment of participants with direct experience of TB treatment, 35–55-minute interviews, repeated engagement with the transcripts, and detailed manual coding. Auditability/dependability was strengthened by an explicit analytical pathway, a progressively refined codebook, NVivo 12 data management, multi-researcher coding and external review of coded material. Confirmability was enhanced by retaining traceability between participant accounts, meaning units, codes, subthemes and final themes, and by using quotations to show the empirical basis of interpretation. Transferability was supported by detailed description of the Jakarta settings and available participant characteristics, allowing readers to judge relevance to other contexts without implying statistical generalisability.

Reflexivity was recognised as important because qualitative data are co-produced through interaction and interpreted by researchers whose clinical, disciplinary and cultural positions may shape what is noticed and how it is understood ( O’Brien et al., 2014; Tong et al., 2007). The research team comprised nursing and health-science academics and clinicians with qualitative research experience. Field notes and analytic memos supported reflection during data generation and analysis, while external review of coded material and whole-author review of the final themes exposed interpretations to perspectives beyond the primary coding group.

### Ethical considerations

Ethical approval was obtained from the Binawan University Health Research Ethics Committee (REC) (No. 033/KEPK-UBN/I/2023). Before data collection, participants received a comprehensive explanation of the study and provided written informed consent. They were informed of their right to withdraw without unfavourable consequences. Strict confidentiality and anonymity procedures were established because interviews addressed potentially sensitive experiences of illness, rejection, family relationships and discrimination.

Each participant was assigned an alphanumeric code (e.g., P1, P2, P3), and identifiers were removed from transcripts and reported quotations. Audio-recordings and transcripts were stored on a password-protected computer in the first author’s office and were accessible only to the research team. Study materials will be retained for three years and then securely disposed of in accordance with the approved protocol.

## Results

Thirty people receiving TB treatment participated: 12 women and 18 men, aged 25–57 years. Most had been living with TB for at least one year, and all were in the continuation phase of treatment at interview. Four interrelated themes were identified: (1) internalised stigma or self-stigma; (2) family stigma; (3) public/social stigma; and (4) reasons for TB stigmatization. Together, the themes show how TB was experienced not only as a health condition but also as a socially consequential identity. Across participants’ accounts, fear of transmission and misinformation contributed to labelling and avoidance, which could then be internalised as shame or expressed through withdrawal from family and community life.

### Theme 1: Internalised stigma or self-stigma

Participants described internalised stigma as a process through which external judgements became incorporated into their own sense of self. Shame, perceived loss of status and anticipation of rejection altered how some participants understood their place in the community. Internalised stigma was therefore not simply an emotional response to illness; it reflected the absorption of social meanings attached to TB.

***Status loss and shame***

Some participants described feeling socially diminished after being identified as a person with TB. The language of humiliation and discredit suggested that diagnosis was experienced as a change in social status, with consequences for dignity and belonging.

“ *Yes, I experience a status loss … shame. In society, insult and discredit exist* … *I am insulted. I am considered a TB patient and humiliated. I have been offended. I am sad, and my heart sobs uncontrollably …”* (Participant 4)

This account illustrates how social labelling was internalised as shame and emotional pain. The participant did not describe stigma as an abstract community attitude; it was experienced as an assault on social identity and self-worth.

***Rejection, avoidance and perceived discrimination***

Anticipated and experienced rejection also contributed to self-stigmatization. Participants described becoming highly aware of how others responded to their presence, with avoidance interpreted as evidence that they had become socially unacceptable.

“ *Indeed, those afflicted with tuberculosis experience social rejection. The community disapproves of this illness. It appears that only a few people can accept our condition without rejection. Additionally, a problem is that tuberculosis causes avoidance … I’ve noticed that others tend to avoid my presence …”* (Participant 15)

The account demonstrates the interaction between perceived community judgement and the participant’s own expectations of rejection. The experience of avoidance reinforced a sense of difference from those considered socially ‘normal’.

“… *I experience discrimination* … *We [people with TB] are often subjected to unfair treatment compared with individuals with other medical conditions, such as hypertension and diabetes. Yes, I am a victim of discrimination.”* (Participant 25)

Here, stigma was interpreted comparatively: the participant contrasted TB with non-communicable illnesses and perceived that the TB diagnosis attracted a qualitatively different moral and social response.

### Theme 2: Family stigma

Family relationships were an important site of both potential support and stigmatization. Participants described circumstances in which relatives’ embarrassment, fear or rejection reduced the support available during treatment. Family stigma therefore extended the social consequences of TB into the household and could transform relationships that might otherwise function as key sources of practical and emotional support.

***Reduced family support***

Several participants linked reduced family support to relatives’ feelings of shame. In these accounts, stigma was not confined to the person diagnosed with TB but affected how family members believed they themselves might be perceived.

“ *… My family members do not provide support for me. Occasionally, because of my condition, my parents experience feelings of humiliation* … *my father and mother do not support me. Because … yes, they [family members] experience a feeling of shame.*” (Participant 10)

The quotation illustrates an associative dimension of stigma: perceived family shame translated into reduced support for the person undergoing treatment. This is clinically important because family support can be central to coping with a prolonged treatment trajectory.

***Family rejection***

Participants also described rejection within extended families. Such rejection appeared to persist even when the person with TB was engaged with healthcare, suggesting that treatment itself did not necessarily remove the social meaning attached to the diagnosis.

*“Yes, they are typically rejected* … *Families may find it particularly challenging to convince long-term TB patients to accept their relatives. Because they believe that a person with TB will be disgraced. Then, an individual with TB is rejected or refused.”* (Participant 16)

This account suggests that family stigma may be sustained by anticipated disgrace and social judgement rather than solely by immediate concern about infection.

### Theme 3: Public/social stigma

Public and social stigma was expressed through labelling, withdrawal of support, isolation, exclusion and discrimination. Participants’ accounts indicated that TB could become a durable social label with consequences extending into employment and community participation. Public stigma was therefore experienced both symbolically, through identity and reputation, and materially, through lost opportunities and exclusion.

***Social labelling and employment consequences***

Participants described being labelled by others as a ‘TB’ or ‘ex-TB’ person. The persistence of the label beyond active illness was particularly important because it could affect opportunities after treatment.

“ *… I am stigmatized or labelled by my society* … *I am labelled as a TB … or other as an ex-TB person. Then, if I look for employment, it is challenging for me …*” (Participant 22)

The participant’s description shows how a medical history can become a continuing social identity. The reference to employment demonstrates that stigma may have economic consequences even after treatment.

***Limited social support and isolation***

Some participants described a marked absence of community support and a sense that others wished to distance themselves from them.

“ *Support from them [people in the community] is limited. Fully true* … *I do not have any form of support* … *I have no support whatsoever. Members of the community only have concerns with themselves. No, I do not get support from others at this time. They want me to leave …”* (Participant 21)

The account conveys more than loneliness; it suggests perceived social expulsion, with the participant understanding limited support as an indication that their continued presence was unwelcome.

“ *Yes … without a doubt, this remains there. They isolated me [members of the community]. Consequently, I was angry and had no desire to interact with the residents of my community. I continue to experience [the desire for] vengeance due to my rejection and isolation.*” (Participant 7)

Here, enacted exclusion generated anger and reciprocal withdrawal. The account illustrates how stigma can alter social relationships in both directions: community avoidance produces emotional injury, which can subsequently reduce the participant’s willingness to re-engage.

***Lack of social acceptance and discrimination***

Participants also described broader patterns of rejection and fear within their communities.

“ *People with tuberculosis were excluded from society due to societal rejection. Others reject them. They will avoid individuals who are afflicted. Individuals experience fear because of this [TB] …*” (Participant 4)

Fear was understood as a central mechanism linking the diagnosis to avoidance. In some accounts this progressed from interpersonal distance to overt discrimination.

“ *Individuals are discriminated against due to tuberculosis. Yes, it is true* … *discrimination. Employers are unwilling to rehire an individual with tuberculosis in his or her previous position because he or she is ex-tuberculosis …*” (Participant 12)

This quotation demonstrates the material consequences of stigma: the designation of ‘ex-tuberculosis’ was perceived to restrict return to previous employment and therefore had potential financial as well as social effects.

### Theme 4: Reasons for TB stigmatization

Participants attributed stigma to interconnected deficits in knowledge, fear of infection, concern about transmitting TB to others and the social association of TB with HIV/AIDS. These accounts suggest that stigmatization was not driven by a single misconception; rather, it emerged from the interaction between incomplete biomedical knowledge, emotional responses to contagion and pre-existing stigma attached to other conditions.

***Limited knowledge and misconceptions***

Participants repeatedly linked avoidance to incomplete understanding of TB causation, transmission and curability. Particularly important was the belief that social risk persisted even after treatment or recovery.

“ *Regardless of the extent of treatment administered or the announcement of recovery, people will continue to avoid him out of concern that they may transmit the disease to another. It is because of a lack of knowledge. They do not understand that TB can be cured …”* (Participant 9)

The participant explicitly connected social avoidance with misunderstanding of curability. This distinction is important because it indicates that stigma can persist after the biomedical basis for isolation has changed.

***Fear of infection***

Fear was described as a powerful driver of social distance. Participants understood community responses as motivated by the possibility of acquiring TB, even where the nature or magnitude of risk was not well understood.

“ *The community members are experiencing fear, sir* … *feeling fearful, frightened, or apprehensive. Yes, society is fearful. Indeed, individuals often experience fear and tend to flee from threatening situations. Due to societal fear, those suffering from TB are often neglected.”* (Participant 25)

This account positions fear as a mechanism through which a perceived health threat is translated into neglect of the person experiencing the disease.

***Fear of transmitting TB to others***

Fear was not restricted to community members. Some participants themselves withdrew because of concern that they could harm others, showing how public-health messages about transmission may be internalised in ways that promote social isolation.

“ *… I choose to be alone. I do not like being with another person … She [a mother] informs me not to be close to a small child because she is at risk of contracting TB. Yes, I chose to withdraw from them. I might infect them …*” (Participant 11)

The participant’s withdrawal can be interpreted as simultaneously protective and stigmatizing: concern for others’ safety led to self-isolation and reduction in ordinary social contact.

***Association with HIV/AIDS***

Participants also described fear that TB could be interpreted as evidence of HIV infection. This represents intersecting stigma, whereby the social meaning of one condition is intensified through association with another highly stigmatised diagnosis.

“ *I was not encouraged by family members to undergo HIV testing, as they would blame me. You know, if someone discovered he was positive [HIV/AIDS] … HIV is among the most feared diseases among individuals.”* (Participant 15)

The account suggests that HIV-related stigma may create an additional barrier to testing and disclosure for people with TB. It also illustrates how stigma can operate through association rather than through the TB diagnosis alone.

## Discussion

This study explored how people receiving TB treatment in two Jakarta healthcare settings experienced stigma and stigmatization. Four interconnected findings were identified: internalised stigma, family stigma, public/social stigma, and perceived reasons for stigmatization. Taken together, the findings suggest that TB stigma operates as a multilevel process in which fear and misinformation contribute to social labelling and avoidance; these responses may then be experienced as discrimination, absorbed as shame or anticipated rejection, and reproduced within families and communities. This pattern is consistent with the Health Stigma and Discrimination Framework, which conceptualises stigma as a process connecting drivers and facilitators to stigma marking, manifestations and downstream health and social outcomes ( Stangl et al., 2019).

The first theme demonstrates the internalisation of social judgement. Participants described shame, loss of status and a sense of being treated differently from people with less stigmatised conditions. Internalised stigma has previously been associated with reduced self-esteem, concealment and difficulty engaging with care ( Cremers et al., 2015; Lee et al., 2017). The present findings add contextual depth by showing how these psychological consequences were linked to concrete interpersonal experiences of insult, avoidance and discrimination. Rather than viewing self-stigma as an isolated intrapersonal problem, the findings suggest that it is produced within a social environment that repeatedly communicates that TB marks the person as different or dangerous.

This interpretation is particularly important given recent Indonesian evidence linking TB stigma with depression and poorer quality of life. Fuady et al. (2024) found that moderate TB stigma was common among adults receiving TB care and that stigma was associated with more severe depressive symptoms and lower quality of life. The present qualitative findings help illuminate possible experiential pathways underlying those associations: humiliation, rejection and loss of social identity may accumulate as psychosocial burdens alongside the physical and financial demands of treatment. Screening for psychological distress and stigma within TB services may therefore be justified, particularly where patients describe withdrawal, shame or fear of disclosure.

The second theme demonstrates that stigma extends into family relationships. Families can be critical sources of practical, emotional and treatment support, yet participants described reduced support, shame and rejection. This resembles the concept of family or associative stigma, in which negative meanings attached to a health condition extend to those connected with the affected person ( Park & Park, 2014; Stangl et al., 2019). Indonesian research has similarly linked limited family knowledge with negative responses to people with TB ( Lay et al., 2021). The implication is that interventions directed only at the individual patient may be insufficient. Family-inclusive education and counselling should address transmission risk, curability and the emotional consequences of rejection while avoiding framing relatives as simply uninformed or blameworthy.

The third theme concerns public and social stigma. Participants described labelling, social exclusion, limited support and employment discrimination. These findings align with qualitative evidence from India, Nigeria, South Africa and other settings where people with TB have described gossip, avoidance, altered social roles and reduced employment or marriage prospects ( DeSanto et al., 2023; Mukerji & Turan, 2018; Oladele et al., 2021). Particularly notable in this study was the persistence of the ‘ex-TB’ label. This suggests that stigma may continue after clinical improvement and that recovery is not solely biomedical. Social recovery may require restoration of occupational opportunity, community participation and a non-stigmatised identity.

Employment discrimination is especially important because TB already carries substantial financial consequences. Loss of work or difficulty returning to employment can intensify the economic burden of treatment and may reinforce concealment of diagnosis. Indonesian researchers have developed a workplace TB-stigma measure precisely because workplace attitudes represent an important but under-recognised dimension of TB control ( Soemarko et al., 2023). The present findings support the need for employer education, confidentiality protections and clear return-to-work guidance based on infectiousness and fitness rather than diagnostic labels.

The fourth theme identifies perceived mechanisms driving stigma. Participants repeatedly referred to limited knowledge about TB, fear of infection, concern about infecting others and association with HIV/AIDS. Fear of contagion is consistently reported across TB-stigma research and is understandable given that TB is transmissible; however, stigma arises when infection-control concerns are generalized into devaluation of the person or persist beyond the period of meaningful infectious risk ( Chang & Cataldo, 2014; Courtwright & Turner, 2010). Effective stigma reduction therefore requires communication that is both accurate and nuanced. Messages that emphasise transmissibility without equal emphasis on curability, effective treatment and changing infectiousness may inadvertently reinforce fear.

The finding that participants sometimes withdrew to protect others also complicates a simple distinction between enacted and internalised stigma. Self-isolation may reflect responsible concern, anticipated judgement, internalised fear or several of these processes simultaneously. This underscores the importance of person-centred communication. People with TB require clear, individualised guidance about when precautions are necessary, when ordinary social contact can safely resume, and how to explain this to family and community members. Such communication may reduce unnecessary isolation without minimising legitimate infection-control requirements.

The association between TB and HIV/AIDS in participants’ accounts illustrates intersecting stigma. Wouters et al. (2020) described the dynamics of double stigma within the HIV-TB co-epidemic, showing that one diagnosis can alter how the other is perceived and managed. In the present study, fear of being associated with HIV was linked to reluctance around HIV testing and anticipated blame. Integrated TB/HIV services should therefore consider confidentiality and stigma explicitly, ensuring that co-testing is framed as routine clinical care rather than a marker of presumed behaviour or identity.

These findings also have direct implications for stigma-reduction interventions. Information campaigns remain necessary but are unlikely to be sufficient where stigma is embedded in family relationships, community norms, workplaces and internalised self-concept. Reviews of TB stigma interventions have called for multilevel approaches combining education with social contact, psychosocial support and structural change ( Foster et al., 2022; Mottershead et al., 2024; Nuttall et al., 2022). Encouragingly, recent Indonesian work has co-developed a community-based, peer-led psychosocial intervention incorporating individual psychological assessment and counselling, monthly peer-led group counselling, individual peer support and community TB Talks ( Fuady et al., 2025). The present findings provide qualitative support for each of these components: counselling may address shame and distress; peer support may counter isolation; family and community dialogue may correct misconceptions; and broader advocacy may address discriminatory practices.

At service level, TB programmes should therefore consider integrating routine assessment of stigma and psychological distress into person-centred care, establishing confidential referral pathways for psychosocial support, involving families where appropriate and desired by the patient, and using peers or TB survivors as credible sources of recovery-oriented information. At community level, communication should differentiate infectiousness from identity and explicitly address the persistence of stigma after treatment. At organisational and policy levels, attention is required to confidentiality, workplace discrimination and protection of people who have completed or are successfully receiving treatment. These recommendations arise directly from the four themes rather than from epidemiological background introduced after the findings.

### Strengths, limitations and contribution to practice

A principal strength of this study is its focus on the perspectives of people receiving TB treatment themselves. The qualitative descriptive design allowed participants to describe stigma across personal, family and community domains in language closely connected to everyday experience. Recruitment from both hospital and primary-care settings provided perspectives from two points in the urban TB-care pathway. The use of semi-structured interviews, contextual field notes and iterative content analysis generated a dataset capable of demonstrating how stigma is experienced and interpreted rather than merely measuring its prevalence. The findings also have strong contemporary relevance because recent Indonesian research has identified substantial co-occurring stigma, depression and unmet psychosocial-support needs ( Fuady et al., 2024).

Several limitations should nevertheless be recognised. First, participants were recruited from only two urban healthcare facilities in Jakarta; the findings should therefore not be treated as representative of Indonesia nationally, particularly given the country’s cultural, geographic and health-system diversity. Second, purposive sampling prioritised experiential depth rather than population representativeness. Although age, sex, treatment duration and treatment context were available, occupation, living environment and setting-specific participant counts were not available in the material supplied for this revision, limiting finer assessment of transferability. Third, stigma is socially sensitive, and interview accounts may have been influenced by recall, social desirability, interview context and participants’ perceptions of the researchers. Fourth, the study involved translation from Bahasa Indonesia into English. Although analysis was undertaken in the original language and translations were independently reviewed for clarity and fidelity, some linguistic or cultural nuance may nevertheless have been altered. Finally, the study relied on self-reported experiences and did not independently verify the reported behaviour or attitudes of family, community members or employers.

Despite these limitations, the study contributes by integrating four dimensions of stigma within a single qualitative account: internalised stigma, family stigma, public/social stigma and participants’ explanations for stigmatization. This helps move the literature beyond documenting whether stigma exists towards understanding how fear, misinformation, labelling and social relationships interact. The findings support a multilevel model of response that combines accurate communication with psychological support, family engagement, peer-led approaches, community dialogue and protection from discrimination.

## Conclusion

For participants receiving TB treatment in these two Jakarta settings, stigma was experienced as more than a negative attitude. It operated through shame and status loss, family rejection, community isolation, persistent social labelling and discrimination, and was sustained by misinformation, fear of infection and association with HIV/AIDS. These processes could remain socially consequential even when treatment had begun or recovery was anticipated.

TB programmes should therefore conceptualise stigma reduction as part of person-centred care rather than as an optional educational activity. Accurate communication about transmission and curability should be combined with psychosocial assessment, peer support, family-inclusive interventions, community engagement and measures to prevent employment and social discrimination. Future research should evaluate how these multilevel interventions influence stigma, mental health, treatment engagement and social recovery across diverse Indonesian settings.

## Data Availability

Participant data contain sensitive personal information, and unrestricted public sharing could compromise confidentiality and anonymity. In accordance with the approved ethics arrangements, access may be considered for qualified researchers with a legitimate academic purpose under conditions that protect participant privacy. Requests should be submitted in writing to the corresponding author, Dr Aan Sutandi ( aan@binawan.ac.id).
