# Supplementary material for: TB Stigma and Stigmatization Experienced by People with Tuberculosis: A Qualitative Study in Jakarta, Indonesia
Source: F1000Res. 2026 Sep 17;15:1130. Originally published 2026 Jul 10. [Version 2] doi: 10.12688/f1000research.177509.2 (PMC13615351; doi:10.12688/f1000research.177509.2)
Supplement: Peer review history

# Reviewer report: Reviewer response for version 2

**DOI:** 10.5256/f1000research.209100.r522089

**Published:** 2026-09-25

**Authors:** Tintin Sukartini

**Affiliations:** 1 Universitas Airlangga, Surabaya, East Java, Indonesia

Thank you for your efforts in addressing the comments. We appreciate the time and attention you devoted to revising the work. All of the comments have been addressed thoroughly and appropriately.

Is the work clearly and accurately presented and does it cite the current literature?

Partly

If applicable, is the statistical analysis and its interpretation appropriate?

Partly

Are all the source data underlying the results available to ensure full reproducibility?

Partly

Is the study design appropriate and is the work technically sound?

Yes

Are the conclusions drawn adequately supported by the results?

Partly

Are sufficient details of methods and analysis provided to allow replication by others?

Partly

Reviewer Expertise:

Qualitative study

I confirm that I have read this submission and believe that I have an appropriate level of expertise to confirm that it is of an acceptable scientific standard.

# Reviewer report: Reviewer response for version 2

**DOI:** 10.5256/f1000research.209100.r522090

**Published:** 2026-09-21

**Authors:** Shwikar Othman

**Affiliations:** 1 University of South Australia, Adelaide, South Australia, Australia

I would like to thank the authors for their careful attention to my previous comments and queries. The manuscript has been substantially revised, and the amendments have contributed to greater clarity, coherence, and overall improvement in the presentation of the study.

 With regard to the Results section, I note that the subthemes remain presented as separate headings and have not been fully integrated into the main narrative. I would encourage the authors to consider removing the individual subtheme headings and incorporating the relevant subthemes more explicitly within the descriptive text. Integrating these elements into the narrative may enhance the flow and readability of the Results section, while also providing a more cohesive and consistent presentation of the findings.

 Overall, the revisions have strengthened the manuscript, and addressing this remaining point would further improve the clarity and organisation of the results section.

Is the work clearly and accurately presented and does it cite the current literature?

Yes

If applicable, is the statistical analysis and its interpretation appropriate?

Yes

Are all the source data underlying the results available to ensure full reproducibility?

Yes

Is the study design appropriate and is the work technically sound?

Yes

Are the conclusions drawn adequately supported by the results?

Yes

Are sufficient details of methods and analysis provided to allow replication by others?

Partly

Reviewer Expertise:

Public health, qualitative research, quantitative research, mixed methods, midwifery, Indigenous health, meta-analysis, meta-synthesis, and meta-aggregation.

I confirm that I have read this submission and believe that I have an appropriate level of expertise to confirm that it is of an acceptable scientific standard.

# Reviewer report: Reviewer response for version 1

**DOI:** 10.5256/f1000research.195743.r504494

**Published:** 2026-08-10

**Authors:** Tintin Sukartini

**Affiliations:** 1 Universitas Airlangga, Surabaya, East Java, Indonesia

Thank you for the opportunity to review the manuscript entitled "TB Stigma: Stigma and Stigmatization Experienced by People with Tuberculosis: A Qualitative Study in Indonesia". I have several comments that the authors may wish to consider in order to improve the clarity of the manuscript.

1. The title contains two colons, which in my view requires further attention. English editing (not proofreading) is needed to improve the clarity and readability of the manuscript overall.
2. What is the distinction between stigma and stigmatisation in this study? The authors highlight both terms repeatedly in the title, and the conceptual difference between them should be made explicit.
3. In the background section of the abstract, please state the gaps identified in previous studies so that the novelty of the present work can be addressed through the study purpose.
4. The study was conducted in two urban areas of Jakarta. Can these findings reasonably be generalised to the national level, as the title implies? Please reconsider the wording of the title accordingly.
5. The introduction is, in my view, too long and focuses excessively on prevalence. Paragraphs 1 and 2 could be condensed into a single paragraph. In addition, in the final paragraph I would encourage the authors to present evidence from previous comparable studies, identify those studies explicitly, and then articulate the gaps arising from them. Those gaps should subsequently be resolved by the novelty of the present study. Please revise this section carefully, as it is a critical part of the manuscript.
6. In the methods section, please specify the reporting guideline followed, such as COREQ or SRQR, and structure the methods in accordance with that guideline.
7. Please provide the inclusion and exclusion criteria in detail.
8. In the results section, please provide detailed information on participant demographics, ideally presented in a table.
9. Please elaborate the description accompanying each quotation and provide an interpretation of the meaning conveyed by each quotation.
10. I have a major concern regarding the analytical process. As this study employed content analysis, please provide a table demonstrating how the analysis was conducted, from initial coding through to the derivation of the final themes.
11. The discussion is too lengthy. Please make it more concise and focus it on the five themes. The discussion may also be strengthened by drawing on a relevant theoretical framework, if appropriate.
12. What are the strengths of this study? These should be stated explicitly alongside the limitations.

Good luck with your study.

Is the work clearly and accurately presented and does it cite the current literature?

Partly

If applicable, is the statistical analysis and its interpretation appropriate?

Partly

Are all the source data underlying the results available to ensure full reproducibility?

Partly

Is the study design appropriate and is the work technically sound?

Yes

Are the conclusions drawn adequately supported by the results?

Partly

Are sufficient details of methods and analysis provided to allow replication by others?

Partly

Reviewer Expertise:

Qualitative study

I confirm that I have read this submission and believe that I have an appropriate level of expertise to confirm that it is of an acceptable scientific standard, however I have significant reservations, as outlined above.

# Response

**Published:** 2026-08-20

**Authors:** Dr. Richard Mottershead

**Affiliations:** College of Health Sciences, University of Sharjah, Sharjah, Sharjah, United Arab Emirates

Thank you for your detailed and constructive review. We have carefully considered each of your recommendations and have substantially revised the manuscript to improve its conceptual clarity, methodological transparency and readability.

 The title has been revised to “TB Stigma and Stigmatization Experienced by People with Tuberculosis: A Qualitative Study in Jakarta, Indonesia”, removing the previous double-colon construction and appropriately locating the findings in Jakarta rather than implying national generalisability. We have also explicitly distinguished stigma from stigmatization in the Introduction. The Abstract now identifies the gap in previous research and more clearly establishes the novelty and purpose of the study.

 The Introduction has been substantially restructured and edited, with excessive epidemiological material reduced and greater emphasis placed on comparable studies, the Indonesian context and the specific knowledge gap addressed by this study. The manuscript has also undergone substantive English-language editing throughout.

 The Methods have been reorganised with reference to COREQ and SRQR and now provide substantially greater detail regarding eligibility, recruitment, participant characteristics, interviewing, transcription, translation, coding, data management, researcher roles, trustworthiness and reflexivity. We have added a participant-characteristics table and, importantly, an analytical pathway table showing the progression from participant meaning units through initial coding and subthemes to the final themes.

 The Results have been rewritten to provide greater interpretation around each quotation. The Discussion has been considerably shortened and focused on the empirical findings, with interpretation strengthened through the Health Stigma and Discrimination Framework.

 We would also like to clarify that the analysis generated four overarching themes rather than five. An inconsistency in the numbering of themes in the previous version may have created this impression; this has now been corrected throughout the manuscript.

 Finally, the revised manuscript explicitly addresses the study's strengths, limitations and contribution to practice.

 Thank you again for your detailed recommendations. We believe they have resulted in a substantially clearer and methodologically stronger manuscript.

 On behalf of the research team - Dr. Richard Mottershead

# Reviewer report: Reviewer response for version 1

**DOI:** 10.5256/f1000research.195743.r504489

**Published:** 2026-08-06

**Authors:** Shwikar Othman

**Affiliations:** 1 University of South Australia, Adelaide, South Australia, Australia

I would like to thank the Editor for the opportunity to review this manuscript and the authors for their work. I hope the comments below are helpful in strengthening the manuscript. Please also find the attached document [ 822869.Sutandi_et_al_2026.pdf ]

 Comments

**1. Introduction**

 - Improve the logical flow by beginning with the burden of tuberculosis (prevalence, impact, and financial burden), followed by the environmental and contextual background.

 - Introduce and define key concepts (e.g., secondary stigma) in the Introduction rather than the Discussion.

 - Remove repeated background information from the Discussion.

**2. Methodology**

 - Justify your choice of a qualitative approach.

 Clearly state the qualitative methodology (e.g., qualitative descriptive, phenomenology, grounded theory) and distinguish it from the data analysis method.

 - Describe how the qualitative content analysis was conducted in this study.

 - Specify who conducted the coding and analysis, their expertise, whether coding was independent, and how disagreements were resolved.

 - Describe data preparation, including transcription, translation, data management, and de-identification procedures.

**3. Participants**

 - Include key participant characteristics (e.g., age, time since TB diagnosis, treatment setting, occupation, and living environment).

 - Clearly describe the inclusion criteria and recruitment process.

**4. Results**

 - Begin each theme with a brief summary before presenting quotations.

 - Integrate subthemes where appropriate to improve readability.

 - Ensure quotations support, rather than replace, the analytical narrative.

**5. Discussion**

 - Reduce repetition and improve logical flow.

 - Focus on interpreting the findings rather than repeating background information or introducing new concepts.

 - Clearly link recommendations to the study findings.

 - Use person-centered language consistently (e.g., "people with TB").

**6. Strengths and Limitations**

 - Include the study's strengths, limitations, and contributions to the literature and practice.

**Overall recommendation**

 The manuscript addresses a major global public health challenge, affecting millions of people worldwide. TB as one of the leading causes of death from an infectious disease, understanding the mental health consequences is essential for informing comprehensive, patient-centered care and improving health outcomes.

 This manuscript has the potential to make a valuable contribution to literature. However, revisions are needed to improve methodological transparency, reporting of qualitative methods and findings, organisation, and consistency before it is suitable for indexing.

Is the work clearly and accurately presented and does it cite the current literature?

Yes

If applicable, is the statistical analysis and its interpretation appropriate?

Yes

Are all the source data underlying the results available to ensure full reproducibility?

Yes

Is the study design appropriate and is the work technically sound?

Yes

Are the conclusions drawn adequately supported by the results?

Yes

Are sufficient details of methods and analysis provided to allow replication by others?

Partly

Reviewer Expertise:

Public health, qualitative research, quantitative research, mixed methods, midwifery, Indigenous health, meta-analysis, meta-synthesis, and meta-aggregation.

I confirm that I have read this submission and believe that I have an appropriate level of expertise to confirm that it is of an acceptable scientific standard, however I have significant reservations, as outlined above.

# Response

**Published:** 2026-08-20

**Authors:** Dr. Richard Mottershead

**Affiliations:** College of Health Sciences, University of Sharjah, Sharjah, Sharjah, United Arab Emirates

Dear Reviewer, 

 Thank you for your thoughtful and constructive review and for recognising the potential contribution of this study. We have undertaken a substantial revision of the manuscript in response to your recommendations.

 The Introduction has been reorganised to provide a clearer progression from the contemporary burden and wider impact of tuberculosis to the social and contextual dimensions of stigma. Key concepts, including internalised and secondary/associative stigma, are now introduced earlier in the manuscript.

 We have particularly strengthened the methodological reporting. The revised manuscript now explicitly identifies and justifies the qualitative descriptive methodology and distinguishes this from the inductive qualitative content analysis used to analyse the data. We have substantially expanded the description of participant eligibility and recruitment, interviewing, transcription and translation, coding and theme development, researcher involvement, data management, de-identification, trustworthiness and reflexivity. Participant characteristics are now presented more clearly, and an analytical pathway table demonstrates how meaning units progressed through coding and abstraction to the final themes.

 The Results have also been extensively revised. Each theme is now introduced with an analytical summary, and participant quotations are embedded within and interpreted through the analytical narrative rather than being presented as findings in isolation. The Discussion has been shortened and refocused on interpretation of the findings, with recommendations explicitly linked to the themes and person-centred terminology used consistently throughout.

 Finally, we have added a dedicated section addressing the study's strengths, limitations and contribution to practice.

 We greatly appreciate your comments. They prompted important revisions that we believe have substantially improved the methodological transparency, analytical depth and clinical relevance of the manuscript.

 On behalf of the research team - Dr. Richard Mottershead
